# Editorial: Reducing the Harm of Medication–Recent Trends in Pharmacovigilance

**DOI:** 10.3389/fphar.2022.964125

**Published:** 2022-08-30

**Authors:** Elena Ramírez, Miguel González-Munoz, Chanda Kulkarni, Francisco J. de Abajo

**Affiliations:** ^1^ Clinical Pharmacology Department, La Paz University Hospital-IdiPAZ, Faculty of Medicine, Universidad Autónoma de Madrid, Madrid, Spain; ^2^ Immunology Department, La Paz University Hospital-IdiPAZ, Madrid, Spain; ^3^ Free Lancer-Clinical Pharmacologist, Rajeev Gandhi University of Health Sceinces Bangalore, Bangalore, India; ^4^ Clinical Pharmacology Unit, University Hospital Príncipe de Asturias, Madrid, Spain; ^5^ Department of Biomedical Sciences (Pharmacology Section), University of Alcalá (IRYCIS), Madrid, Spain

**Keywords:** adverse drug effect, medication without harm, Pharmacovigilance, Pharmacoepidemiology, causality assessment, adverse drug reaction

Two years ago, we launched this second Frontiers in Pharmacology Research Topic titled *Reducing the Harm of medication: Recent Trends in Pharmacovigilance*, following our first topic “Medication Harm: From Early Identification to Prevention”. Adverse drug reactions (ADRs) are considered to be among the leading causes of morbidity and mortality with very high economic costs. An estimated 5–25% of hospital admissions are due to ADRs, and 6–15% of hospitalized patients experience serious ADRs, causing significant prolongation of hospital stay. Moreover, fatal ADRs are estimated to occur in 0.31% of hospitalized patients [*The Harm of Medication: From Early Identification to Prevention*/Frontiers Research Topic]. Randomized controlled trials are the main premarketing methods used to detect and quantify ADRs but these have numerous limitations, such as the exclusion of patients at higher risk of ADRs (e.g., children, elderly, comorbid, polymedicated), insufficient sample size for the detection of non-frequent ADRs, limited follow-up time for the detection of ADRs after long periods of exposure. In addition, the diagnosis of ADRs is complex and difficult in the Real World due to the non-specificity of ADR symptoms and signs, diagnostic tests are usually absent and a re-challenge is rarely ethically justified. At present, most countries participate in pharmacovigilance through spontaneous reporting systems of ADRs. However, spontaneous reporting systems have limitations such as under-reporting, of up to 90%, and reporting bias, of only ADRs easy to diagnose. Active pharmacovigilance programs may supplement such systems. In this Research Topic, we have had the opportunity to evaluate some of the latest strategies in pharmacovigilance programs to cover the much-needed timely and accurate identification of ADRs. Pharmacovigilance programs assessing ADRs occurring in routine clinical practice can contribute to a better knowledge of the risk profiles of a medication classes. A study using national health insurance data evaluated the association between antidepressant use dose/duration and cardiovascular disease (CVD) risk in patients without a history of CVD as a primary prevention measure Jang et al. The authors of this study found that even at low doses, the use of tricyclic antidepressants was associated with major adverse cardiovascular events. Further, the longer duration of tricyclic antidepressant use correlated significantly with a higher adjusted hazard ratio. In another national population-based retrospective cohort study the authors found that the use of “sleeping pills” was associated with an increased risk of chronic kidney disease. Liao et al.

The use of hospital databases is essential for evaluating the safety of medicines for hospital use and for making decisions for the prevention of serious ADRs. In a retrospective cohort from an Intensive Care Unit (ICU), the effect on survival and neurological outcomes of full-dose epinephrine when used in cardiopulmonary resuscitation was investigated Shi et al. The authors found an inverse association between total epinephrine dosage during resuscitation and worse neurological outcome 3 months after the cardiac event. This study concluded that the early benefit of a medication does not ensure good results in the medium and long term among these patients. The pharmacovigilance system could investigate the functional status and quality of life of patients as indicators of the impact of ADRs. In another retrospective cohort study Kunming et al., the authors aimed to characterize vancomycin-associated acute kidney injury (AKI) in Chinese hospitals. The authors found a higher frequency of concomitant nephrotoxic medication use compared to their use in the USA and that the incidence of vancomycin-associated AKI was higher in patients with a combined use of nephrotoxic medications. The study recommended improving serum creatinine measurement for the AKI diagnosis and the standardization of vancomycin therapeutic drug monitoring. In a multicenter retrospective study exploring the association between mean serum vancomycin trough concentration (VTC) and mortality in Intensive Care Units Hou et al., the authors found that mean VTC was not associated with reduced ICU/hospital related mortality. These results indicate that further investigation is needed to address the issue of vancomycin dosing using mean VTC and support the recommendation of moving from trough levels to a 24-h area under the curve (AUC) to a minimum inhibitory concentration ratio. Another retrospective analysis was conducted on inpatients who received tigecycline treatment from January 2018 to January 2020 Shi et al. Based on the biochemical criteria of Drug-Induced Liver Injury (DILI) and the causality assessment by Roussel Uclaf Causality Assessment Method (RUCAM) using cases with a probable or highly probable causality grading. The author found that tigecycline was associated with liver injury, with a slightly higher incidence (5.7%) than the frequency of “frequent” (5%) defined by the Medical Dictionary for Regulatory Activities. In addition, tigecycline-associated DILI was related to a high maintenance dose, prolonged duration, and a number of concomitant medications with known hepatotoxicity.

It is well known that one of the major research challenges regarding drug safety is the identification of genetic variation of drug targets, drug-metabolizing enzymes, or drug transporters that can clarify the mechanisms of idiosyncratic ADRs. In the case-control study by Frogerini et al., the association between PTGS1 and NOS3 variant alleles and the risk of developing upper gastrointestinal bleeding (UGIB) secondary to complicated peptic ulcer disease was assessed. The authors have shown an increased risk of UGIB in the presence of some genetic variants, regardless of the medication exposure. On the other hand, these genetic variants are said to modify the magnitude of the risk of UGIB in nonsteroidal anti-inflammatory drugs and low-dose aspirin users, since variants rs10306114 and rs5788 are related to a COX-1 with less capacity to produce prostaglandins and reduced platelet aggregation, respectively. Therefore the need for personalized therapy for the prevention of these serious ADRs is emphasized.

Automated screening tools developed from quantitative methods in the large databases of suspected ADR reports allow signal detection to trigger further investigations. Improving pharmacovigilance disproportionality analysis to reduce methodological heterogeneity and substantial variability of results by pre-registering the protocols and presenting a set of secondary and sensitivity analyses rather than a single result may prevent selective reporting of results Khouri et al. In a study based on suspected ADRs from the World Health Organization (WHO) global database (VigiBase) Allouchery et al. the authors conducted a disproportionality analysis of ibrutininb, a Bruton tyrosine kinase inhibitor for the treatment of chronic lymphocytic leukemia, mantle cell lymphoma, Waldenström macroglobulinemia, with respect all other anticancer medications used as the reference group in order to identify of potential safety signals. The authors combined the use of two complementary disproportionality measures (proportional reporting ratio and information component) and found clinically relevant potential safety signals in patients exposed to ibrutinib, mainly ischemic heart diseases, pericarditis, uveitis, retinal disorders, and fractures. Immune checkpoint inhibitors (ICI) may be used to treat aggressive hematologic malignancies, either in refractory or relapsed lymphoma frequently before being treated by allogeneic hematopoietic stem cell transplantation (allo-HSCT) or in relapse after allo-HSCT. Nguyen et al. used the Bayesian estimate of disproportionality analysis of VigiBase to detect a signal of graft vs. host disease (GVHD), with subsequent mortality of 25.8%. This paper confirms previous results obtained from cohort studies on the association between GVHD and ICI. This manuscript is an example that the sum of evidence from different approaches in data analysis (experimental data, clinical trials, spontaneous notifications, case-control studies, cohort studies, and data mining) would allow definitive conclusions and decision-making in pharmacovigilance. Conducting a systematic review and meta-analysis of randomized controlled trials, Man et al. addressed the putative increase risk of malignancies and tuberculosis in patients with spondyloarthritis (SpA) treated with biologics. The authors showed an elevated risk of malignancy in patients with peripheral SpA treated with biologics, especially for IL-17 inhibitors, and an increased risk of tuberculosis in patients with axial SpA treated with anti-TNFα antibody. Since the sparse number of events of malignancy and tuberculosis, these results need to be confirmed by further studies with a larger population and longer follow-ups.

In this Research Topic, we have also evaluated the latest strategies to improve the safety of medication use. In this sense, an integrative systematic review of the practical considerations of Pro Re Nata medication management sought to summarize and integrate the practical considerations of healthcare professionals for the management of Pro Re Nata medications in different healthcare settings. Mardaniet al. On the other hand, high quality understandable, and accessible information helps patients to participate in decision-making about medicines prescribed for them by healthcare professionals. Despite a lot of information available in the media, patients considered patient information leaflets of medical products the most important source of information about medicines after medical prescription. Medina-Córdoba et al. evaluated patients’ cognitive, behavioral, and emotional factors and characteristics of Patient Information Leaflets that can promote appropriate drug use practices. Another study tried to identify factors associated with a lack of awareness of the impact of improperly disposed of medications among the general population in Bandung, Indonesia, and to assess the associations of awareness with medication disposal practices among this population. This study provides results that contribute to a better characterization of some aspects of patients’ medication self-management. Alfian et al.

This topic also contains a case report with an objective to describe a simple, universal, and cost-effective method of microbiome analysis for clinical trials Zdziarski et al. This general method could raise the hypothesis of drug-associated dysbiosis. When testing the method in one of the patients treated with high doses of inhaled corticosteroids, the authors have come across the unexpected finding that severe dysbiosis was followed by seronegative Sjögren’s syndrome.

Finally, a position paper from the ANSM (Agence Nationale de Sécurité du Médicament et des Produits de Santé) proposes some actions to be implemented to reduce the intentional unjustified use of medicines: 1) Early identification of situations in which inappropriate use can lead to a health risk comparing consumption in different countries. 2) Better collective engagement including educational programs and co-construction of action plans and improved communication with stakeholders Vignot et al.

We appreciate the good acceptance of this topic, and we will appreciate good reception among the authors to continue contributing effectively and share their interesting study findings in the second part of this topic (Volume II) [*Reducing the Harm of Medication - Recent Trends in Pharmacovigilance*, Volume II/Frontiers Research Topic].

Finally, we make some suggestions to encourage Pharmacovigilance activities going forward. The first is based on the fact that evidence suggests that individual case safety reports remain a very useful data source for detecting potential new safety issues. We suggest improving diagnostic tools, causality algorithms, and complementary drugs with other *in vitro* tests (pharmacogenetics, pharmacogenomics, pharmaco-immunology, pharmaco-proteomics), in the diagnosis of ADRs. In this sense, the implementation of expert medical specialties in the diagnosis of ADRs (e.g., Clinical Pharmacologists with specific training in the causality of ADRs) could give support and confidence to physicians in the generation of these new drug safety signals from primary and hospital care. On the other hand, electronic health records have proven to be more useful for evaluating problems already detected, allowing the implementation of prevention and early detection tools that minimize the risk of ADRs. Prospective cohort or retrospective observational database studies allow for longer follow-up periods of patients with a much broader range of characteristics, providing valuable means for the detection, quantification, and, where possible, reduction of ADRs, reducing health care costs in the process.

